# Beyond the Curve: Intra-spinal Rib Head Herniation as a Critical Complication of Dystrophic Scoliosis in Pediatric Patients With Neurofibromatosis Type 1

**DOI:** 10.7759/cureus.84841

**Published:** 2025-05-26

**Authors:** Katherine Wei, Kristen Launier, Afshin Aminian, Azam Eghbal, Rahul Nikam

**Affiliations:** 1 Radiology, University of California Irvine School of Medicine, Irvine, USA; 2 Radiology, Children's Hospital of Orange County, Orange, USA; 3 Orthopedics, Children's Hospital of Orange County, Orange, USA

**Keywords:** dystrophic scoliosis, neurofibromatosis type 1, pediatric scoliosis, rib head herniation, spinal fusion

## Abstract

Neurofibromatosis type 1 (NF1) is frequently associated with a multitude of skeletal abnormalities including dystrophic scoliosis. A rare but severe complication of dystrophic scoliosis in NF1 is the herniation of rib heads into the spinal canal, potentially leading to devastating spinal cord compression. We present two pediatric cases of NF1-associated dystrophic scoliosis with intra-spinal herniation of rib heads. Case 1 involves a teenage male with progressive thoracolumbar scoliosis and protrusion of T10 and T11 rib heads into the spinal canal, who underwent successful posterior spinal fusion (T3-L3) with instrumentation, osteotomies, and rib head resection. Case 2 involves a teenage female with progressive thoracolumbar scoliosis and intra-spinal protrusion of T4 and T5 rib heads, who remains neurologically intact despite worsening curvature. Intra-spinal herniation of rib heads is a clinically important complication of NF1-associated dystrophic scoliosis requiring close surveillance. Familiarity with this complication is important as imaging findings may be subtle early on. Surgical management typically involves both spinal fusion and resection of rib heads to prevent neurological compromise, though timing may vary based on symptoms and progression. Multidisciplinary care is essential.

## Introduction

Neurofibromatosis type 1 (NF1) is an autosomal dominant genetic disorder caused by mutations in the *NF1* gene, encoding the tumor suppressor neurofibromin [1]. Loss of function leads to dysregulated cell growth, predisposing individuals to various tumors and systemic manifestations [1]. The 2021 consensus diagnostic criteria require fulfilling specific clinical or genetic findings [2]. While NF1 diagnosis is often clinical, genetic testing can be confirmatory [2,3]. Management involves lifelong surveillance as per guidelines from organizations such as the American Academy of Pediatrics (AAP) and the Children’s Tumor Foundation [4].

Spinal deformities are common in NF1, occurring in approximately 10% of patients [5]. These can be non-dystrophic, resembling idiopathic scoliosis, or dystrophic, characterized by severe, rapidly progressive curves often associated with vertebral abnormalities such as posterior scalloping, rib penciling, and foraminal enlargement [6]. Dystrophic scoliosis frequently presents early and carries a higher risk of complications [6,7]. A specific and concerning feature of dystrophic scoliosis in NF1 is the potential for rib heads to dislocate and protrude into the spinal canal, particularly at the curve's apex [7,8]. Here, we present two pediatric cases of NF1 with dystrophic scoliosis complicated by intra-spinal rib head protrusion.

## Case presentation

Case 1

**Figure 1:**
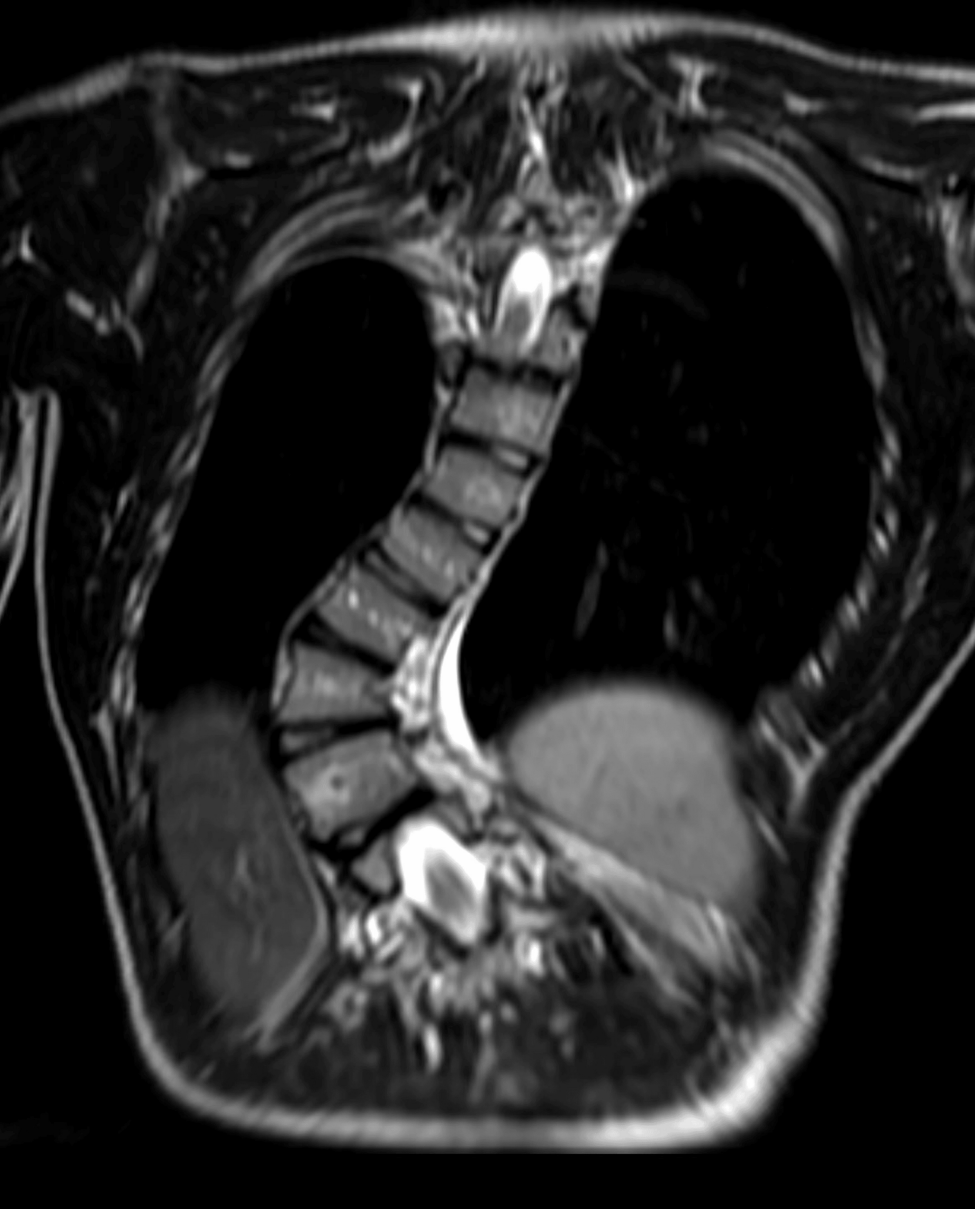
Coronal T2-weighted image of the thoracic spine showing dextrocurvature of the thoracic spine.

**Figure 2:**
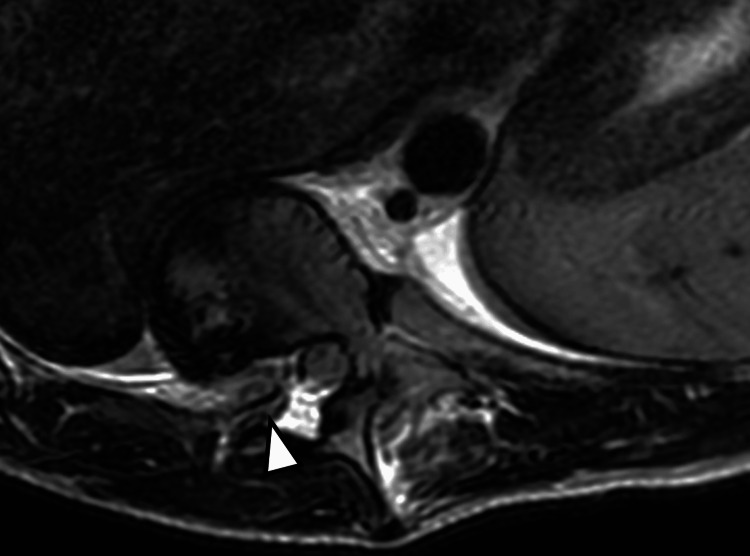
Axial T2-weighted image through T10-T11 level showing protrusion of the right T11 rib head through the neural foramen into the spinal canal. There is no cord compression or signal abnormality at this level. Note that findings of rib herniation can be subtle on MRI, and close attention and familiarity with this condition are essential to identify this finding, particularly in patients with NF1-associated dystrophic scoliosis.

**Figure 3:**
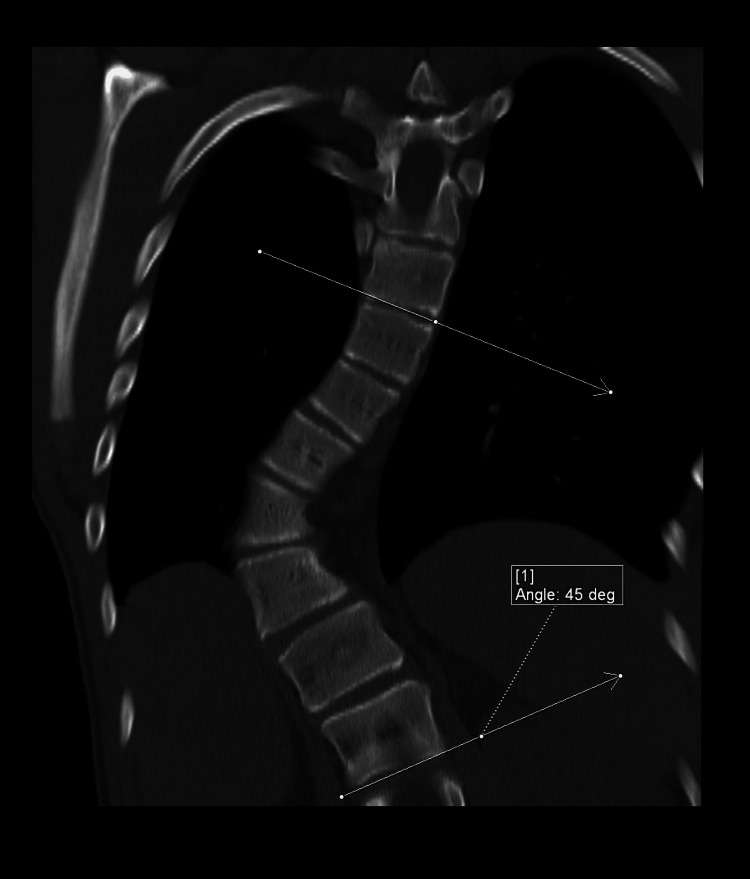
Reconstructed coronal CT image of the thoracic spine in bone kernel showing dextrocurvature of the thoracic spine with its apex at T10-T11. The Cobb angle measured 45 degrees.

**Figure 4:**
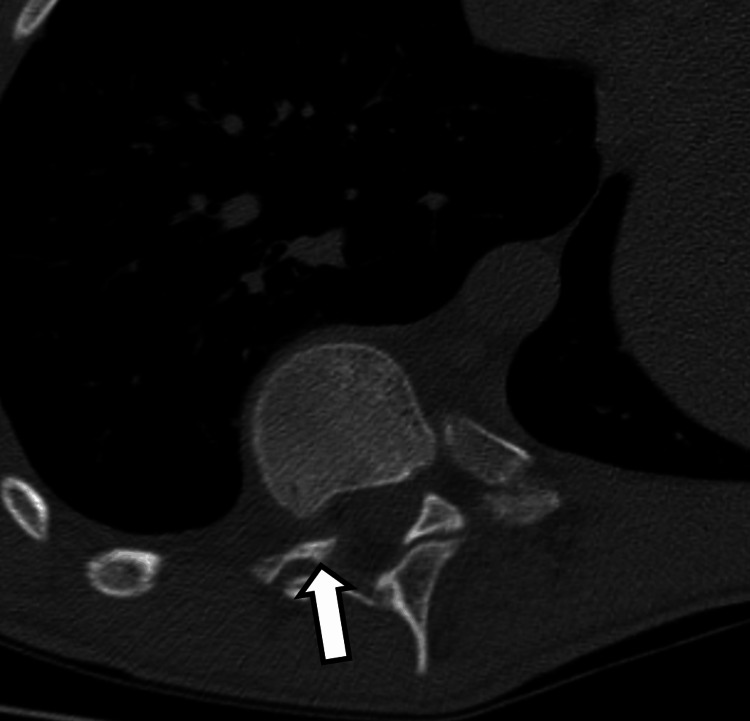
Axial CT image in bone kernel through T9-T10 neural foramen showing protrusion of the pointed head of the rib through the foramen into the spinal canal (arrow).

**Figure 5:**
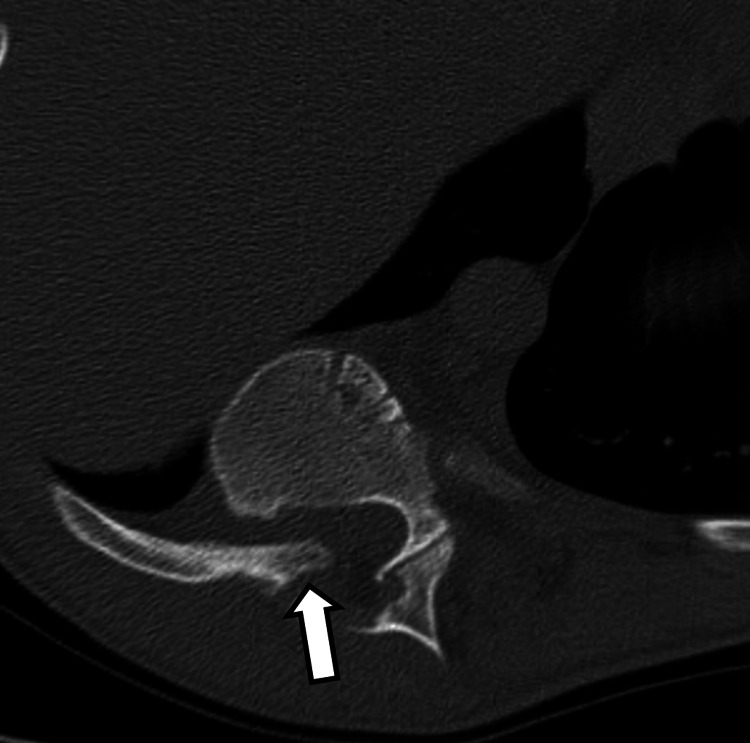
Axial CT images in bone kernel through T10-T11 neural foramen showing protrusion of the pointed head of the rib through the foramen into the spinal canal (arrow).

A 14-year-old male with a clinical diagnosis of NF1 and Chiari 1 malformation presented with progressive dystrophic right thoracolumbar scoliosis. He did not endorse neck pain, sensory changes, motor deficits, bowel/bladder incontinence, dyspnea at rest, chest tightness, restless sleep, morning headaches, or daytime sleepiness. Magnetic resonance imaging (MRI) of the spine was performed, which showed marked thoracic dextrocurvature with apex at T10-T11. At the apex of the curvature, there was herniation of pointed, dysmorphic, right T10 and T11 rib heads into the spinal canal through enlarged T9-T10 and T10-T11 neural foramen, with no significant mass effect on the spinal cord (Figures 1, 2). Computed tomography (CT) of the thoracic spine obtained for evaluation of the osseous anatomy confirmed the findings (Figures 3-5). The Cobb angle measured 45 degrees. Also, there were additional stigmata of NF1 including dural ectasia, and scalloped vertebrae. Due to progressively worsening scoliosis and spinal canal impingement by the rib heads, the patient underwent posterior spinal fusion from T3 to L3 with instrumentation, apical osteotomies, bone grafting, T11 hemilaminectomy, spinal cord decompression, and resection of the protruding rib head.

Case 2

**Figure 6:**
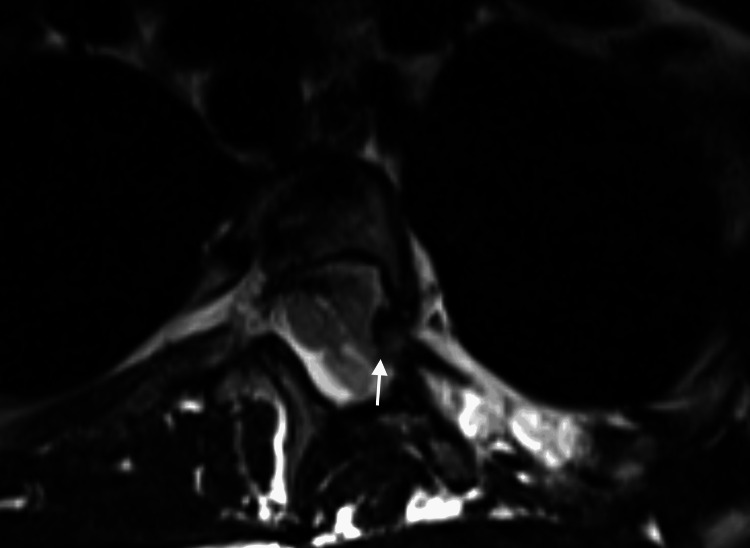
Axial T2-weighted image through the T4-T5 level showing protrusion of the left T5 rib head through the neural foramen into the spinal canal (arrow). There is no cord compression or signal abnormality at this level. Note extensive CSF pulsation artifacts within the thecal sac.

**Figure 7:**
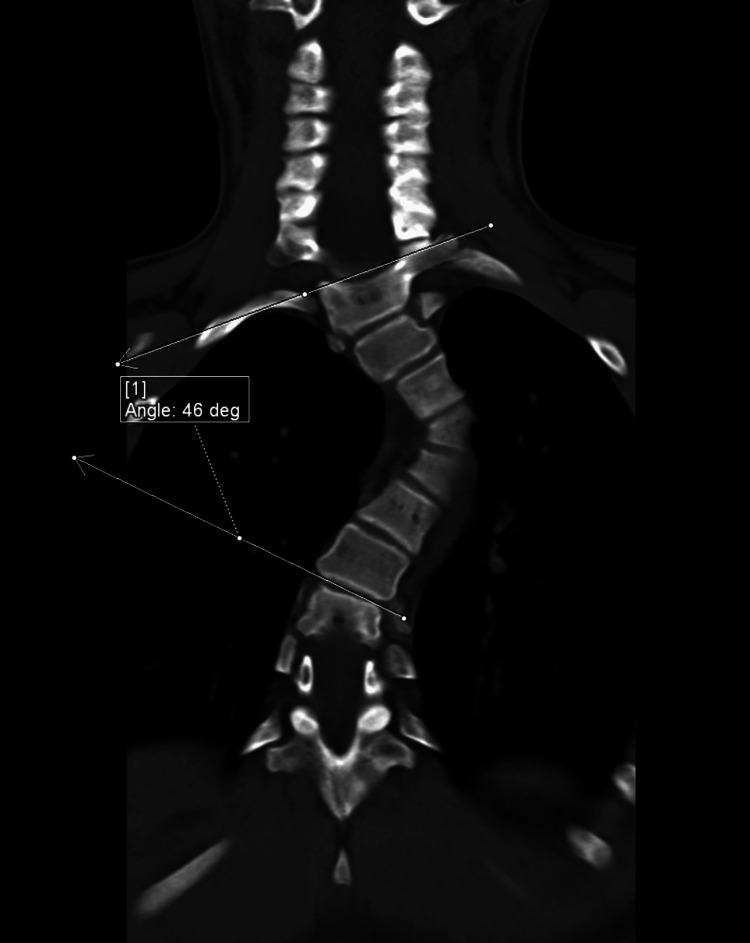
Reconstructed coronal CT image of the thoracic spine in bone kernel showing levocurvature of the thoracic spine with its apex at T4. The Cobb angle measured 46 degrees.

**Figure 8:**
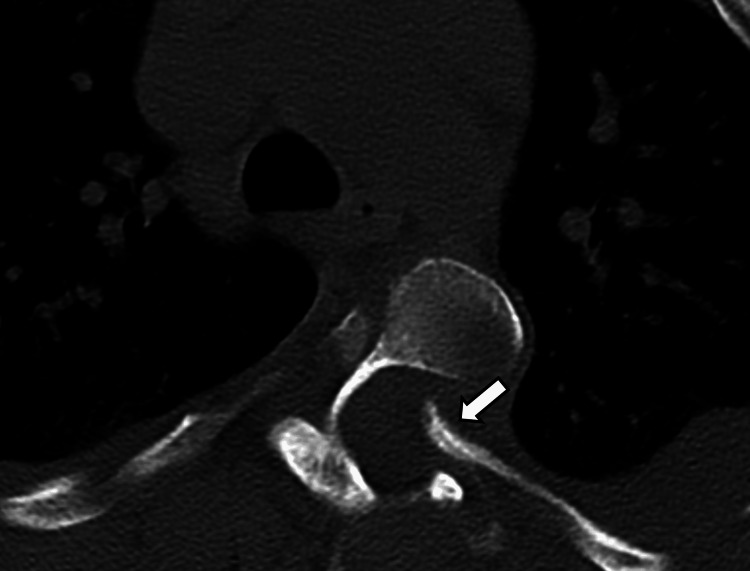
Axial CT image in bone kernel through the T4-T5 neural foramen showing protrusion of the pointed head of the rib through the foramen into the spinal canal (arrow).

**Figure 9:**
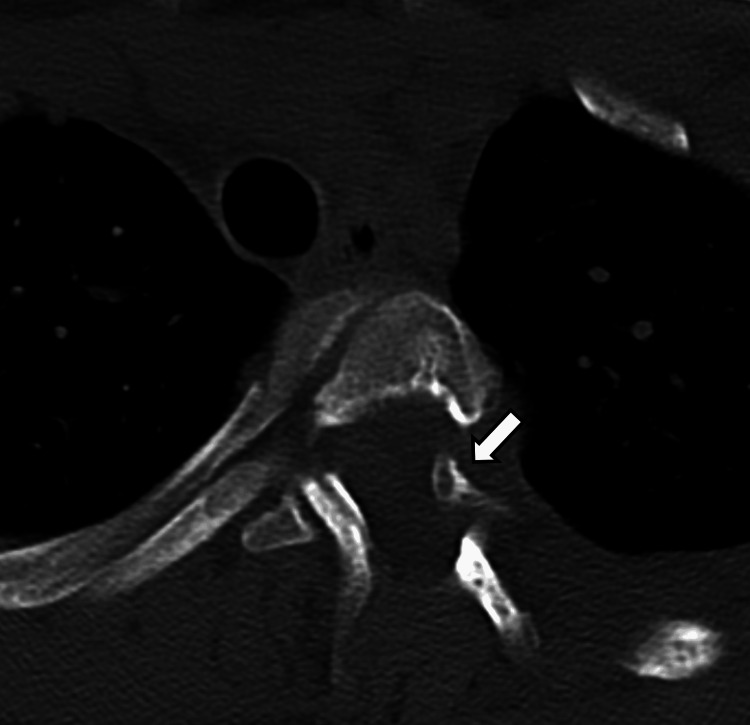
Axial CT images in bone kernel through the T3-T4 neural foramen demonstrate protrusion of the pointed head of the rib through the foramen into the spinal canal (arrow).

A 12-year-old female with NF1 presented with progressive thoracolumbar levoscoliosis with its apex at T4. There were no complaints of neck pain, sensory changes, motor deficits, or bowel/bladder incontinence, but the patient reported occasional back pain, and recurrent pain and numbness in the left foot. There were no complaints of dyspnea at rest, chest tightness, restless sleep, morning headaches, or daytime sleepiness. An MRI of the spine was performed, which showed marked upper thoracic levocurvature with apex at T4. At the apex of the curvature, there was herniation of pointed rib heads into the spinal canal through enlarged T3-T4 and T4-T5 neural foramen (Figure 6). There was no significant mass effect on the spinal cord. A CT of the thoracic spine was obtained for delineation of the osseous anatomy, which confirmed the findings Figures 7-9). The Cobb angle measured 46 degrees. She was managed initially with bracing for scoliosis, but compliance was limited due to skin irritation. Orthopedic evaluation recommended surgery due to scoliosis progression (~10% worsening) and concern for rib encroachment upon the spinal canal.

## Discussion

NF1 is an autosomal dominant genetic disorder caused by mutations in the NF1 gene, located on chromosome 17q11.2 [1]. This gene encodes neurofibromin, a tumor suppressor protein that regulates the RAS-MAPK pathway, controlling cell proliferation [1]. The loss of neurofibromin function leads to dysregulated cell growth, predisposing individuals to neurofibromas, plexiform neurofibromas, optic pathway gliomas, and malignant peripheral nerve sheath tumors [1]. Additionally, NF1 can cause vascular abnormalities, skeletal dysplasia (such as scoliosis and tibial dysplasia), cognitive impairment, and hypertension due to renal artery stenosis or pheochromocytomas [1]. The clinical presentation varies widely, with approximately 50% of NF1 cases resulting from spontaneous mutations [2].

The 2021 consensus diagnostic criteria for NF1 require at least two of the following: six or more café-au-lait macules, axillary or inguinal freckling, two or more neurofibromas or one plexiform neurofibroma, optic glioma, two or more Lisch nodules, a distinctive osseous lesion, a first-degree relative with NF1, or a confirmed pathogenic NF1 variant by genetic testing [4]. Diagnosis is primarily clinical, but genetic testing is recommended for uncertain cases or family planning. Neuroimaging is indicated for symptomatic patients, particularly those with headaches, neurological deficits, or suspected intracranial tumors. Orthopedic evaluations are critical for detecting early skeletal deformities, particularly scoliosis, which can progress rapidly in NF1.

Spinal deformities in NF1 patients can be categorized into dystrophic and non-dystrophic types. The prevalence of spinal deformities in NF1 is estimated to be approximately 10%, with early onset scoliosis being particularly aggressive [9]. NF1 patients with dystrophic spinal deformities present with severe scoliosis requiring surgical correction, often complicated by infiltrating plexiform neurofibromas. Non-dystrophic curves resemble typical idiopathic scoliosis in their presentation and natural history, typically exhibiting gradual progression and responding to conventional scoliosis management, such as bracing and spinal fusion [10]. Dystrophic curves, in contrast, are characterized by their severe and rapidly progressive nature and result from vertebral and paravertebral abnormalities, including vertebral scalloping, rib penciling (width of the rib is smaller than the narrowest portion of the rib), and foraminal enlargement due to neurofibromas or dural ectasias [10]. These dystrophic deformities often present in early childhood and progress aggressively, leading to kyphoscoliosis, vertebral instability, and potentially significant neurological impairment. Dystrophic scoliosis in NF1 is usually marked by a short-segment, sharply angled curve, often accompanied by wedging and scalloping of the vertebral bodies [8]. Additional features may include vertebral body rotation, enlargement of the intervertebral foramina, and rib head tapering. These structural abnormalities increase the risk of intra-spinal rib head dislocation, making this a relatively specific finding in NF1 patients [11].

In a review of four NF1 patients conducted by Ton et al., each patient presented with a single rib head dislocated into the spinal canal at the convex apex of the scoliotic curve, typically in the mid- to lower thoracic spine. Two of these patients exhibited symptoms of spinal cord compression including mild lower limb hyperreflexia, right foot weakness, hyperreflexia, and ankle clonus, while the other two were asymptomatic [11]. While there is no clear consensus regarding the treatment of intra-spinal rib head dislocation in NF1 patients, rib head excision along with posterior spinal fusion is generally considered the standard of care to prevent spinal cord impingement. Each of our patients had two ribs protruding into the spinal canal, at the apex of the dystrophic curve. Although our first patient was asymptomatic for rib herniation, the risk of spinal cord impingement remained. Our second patient did endorse recurrent left foot pain and numbness. A case study by Khoshhal and Ellis documented a case of paraparesis several weeks after posterior spinal fusion without concurrent correction of rib head protrusion into the spinal canal, emphasizing the need for rib head resection. After anterior decompression with resection of the rib was carried out, there was rapid improvement in the patient’s neurological symptoms [12].

Treatment approaches vary depending on the severity and type of spinal deformity. Non-dystrophic curves can be managed conservatively with bracing and observation if progression is mild. Surgical intervention may be required in cases of significant curvature or rapid progression due to their high risk of complications. Unlike in cases of progressive, non-dystrophic scoliosis where the risk is primarily related to deformity magnitude and long-term functional issues, in NF1-associated dystrophic scoliosis, the presence of intra-spinal herniation of dysmorphic, pointed rib head potentially impinging upon the spinal cord adds a critical factor that significantly impacts the decision-making process regarding the surgical timing and approach. The preferred surgical technique is anterior and posterior spinal fusion with segmental instrumentation, which provides greater mechanical stability and reduces the risk of pseudarthrosis [10]. Bracing is largely ineffective for dystrophic curves due to the underlying structural weaknesses in the vertebrae [10]. There are significant complications associated with surgical intervention for dystrophic curves in NF1 patients, including high rates of pseudarthrosis, implant failure, and increased risk of neurologic complications [5]. The presence of paraspinal neurofibromas and dural ectasia further complicates surgical planning and execution. Therefore, a multidisciplinary approach, involving neurosurgeons, orthopedic specialists, and genetic counsellors, is recommended for optimal patient management.

The cases illustrated here depict the clinically significant and potentially ominous complication of intra-spinal rib head herniation associated with dystrophic scoliosis in patients with NF1. Dystrophic curves in NF1 are distinct from non-dystrophic curves and idiopathic scoliosis due to underlying osseous abnormalities and association with neurofibromas, leading to rapid progression and instability [6]. Rib head dislocation into the spinal canal is a relatively specific finding in NF1-related dystrophic scoliosis [7,8].

Overall, NF1 is a multisystem disorder requiring multidisciplinary care and lifelong monitoring. Early recognition and management of skeletal, vascular, and neurological abnormalities are essential to optimizing patient outcomes. As research continues to refine diagnostic and therapeutic strategies, continued documentation of cases will contribute to improved evidence-based guidelines for NF1 management.

## Conclusions

Intra-spinal rib head protrusion is a severe potential complication of dystrophic scoliosis in pediatric patients with NF1. Careful radiological assessment and multidisciplinary management are essential. Surgical intervention including rib head resection should be strongly considered in cases of intra-spinal protrusion to prevent neurological compromise. Continued surveillance is critical for all NF1 patients with spinal deformities. The cases detailed serve as strong examples reinforcing that the decision for surgery in NF1 dystrophic scoliosis with rib head herniation is driven by the neurological risk posed by the bony impingement rather than solely by curve magnitude or typical age-based guidelines for scoliosis. This is a crucial point for clinicians encountering this specific presentation.
